# Dietary Control of Ganglioside Expression in Mammalian Tissues

**DOI:** 10.3390/ijms21010177

**Published:** 2019-12-26

**Authors:** Tetsuya Okuda

**Affiliations:** Bio-Design Research Group, Bioproduction Research Institute, National Institute of Advanced Industrial Science and Technology (AIST), Tsukuba 305-8566, Japan; t-okuda@aist.go.jp; Tel.: +81-50-3648-6162

**Keywords:** ganglioside, glycosphingolipid, ketogenic diet, low-carbohydrate diet, epilepsy

## Abstract

Gangliosides are series of glycosphingolipids containing sialic acids in the oligosaccharide portion in mammalian cells. Gangliosides are a component of cellular membranes and play roles in modulating membrane function and the activity of membrane proteins. Abnormal expression and metabolism of gangliosides lead to the onset of several conditions in humans, such as neurologic diseases, diabetes, and cancer. A number of studies have been carried out to date to investigate the role of gangliosides in these diseases, and the effect of diet on tissue expression of gangliosides has recently become a topic of interest in this field. As gangliosides are degraded in the intestinal tract, ingested food-derived gangliosides are not directly absorbed into tissues in vivo, but the degradation products can be absorbed and affect ganglioside expression in the tissues. Recent studies have also shown that the expression of gangliosides in tissue cells can be indirectly induced by controlling the expression of ganglioside metabolism-related genes via the diet. These results indicate that dietary control can regulate the expression levels of gangliosides in tissues, which is expected to play a role in preventing and treating ganglioside-related diseases. This review introduces recent studies on the effect of diet on the expression of gangliosides in tissues, with a focus on our findings.

## 1. Introduction

Glycosphingolipids are amphipathic molecules composed of oligosaccharides and ceramides and function as a component of cellular membranes in mammalian cells. The oligosaccharide and ceramide portion of glycosphingolipids exhibit structural diversity, and various molecular species of glycosphingolipids have been defined based on differences in their oligosaccharide structures in mammalian cells [1]. ‘Ganglioside’ is a general term to describe an acidic glycosphingolipid containing sialic acid in the oligosaccharide portion, and because it is abundant in ganglia, it was designated as ganglioside.

Gangliosides interact with co-localized proteins in cellular membranes and modulate cellular functions, particularly signal transduction [1,2]. An optimal level of ganglioside expression is required to maintain membrane integrity [3]. Indeed, abnormal expression of gangliosides in tissue cells is associated with a variety of diseases, such as diabetes [4], neurologic diseases [5], and cancer [6]. Thus, a number of studies have been conducted to enhance understanding of the biosynthesis and metabolism of gangliosides as new targets for the prevention and treatment of these diseases.

Little is known regarding the effect of diet on ganglioside expression in vivo; thus, several investigations are now underway to address this issue. The results of related previous studies indicate that gangliosides are degraded in the intestinal tract, and ingested food-derived gangliosides are thought to be indirectly absorbed in the tissues in vivo [7]. Ingestion of saccharides and lipids, which are components of gangliosides, can affect their tissue expression levels [7,8,9]. Furthermore, a recent study demonstrated that diets/foods that affect the expression of ganglioside metabolism-related genes alter ganglioside metabolism in tissue cells as well as ganglioside expression levels [10].

In this review, we comprehensively discuss recent related studies examining the effect of diet on the expression of gangliosides in tissue cells, with a focus on our research results.

## 2. Structures and Molecular Species of Gangliosides

**Figure 1:**
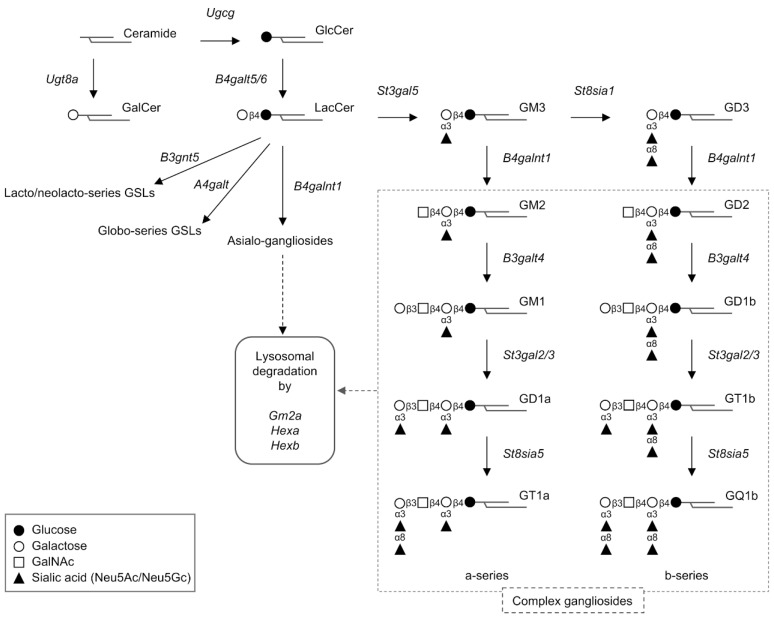
Schematic illustration of the primary metabolic pathway of mammalian gangliosides. Genes encoding proteins involved in ganglioside synthesis and degradation are shown in italics. Solid-line arrows indicate synthetic pathway. Dotted-line arrows indicate degradation pathway.

Gangliosides are produced by animal cells, including those of humans and mice. Figure 1 shows the structures and synthesis pathways of the major molecular species of gangliosides produced in mammalian cells. Gangliosides are composed of a series of glycosphingolipids, which include a number of molecular species defined based on differences in oligosaccharide structure. Ceramides (sphingoid base and fatty acid), which are associated with the immunogenic activity of glycosphingolipids, also exhibit structural diversity [11,12,13].

Each molecular species of ganglioside is expressed differently in different animal species and cell types, and the oligosaccharide portion exhibits antigenicity on the cell surface. As different oligosaccharide structures of gangliosides have different immunogenicity, they can be used as specific antigens to differentiate animal species and cell types [13,14]. Moreover, the oligosaccharide structure can change depending on the differentiation or pathologic state of a cell. These oligosaccharides are known as differentiation or pathologic cell markers.

The diversity of ganglioside structures is determined by the ganglioside metabolism-related genes expressed in the cell [1,2]. In particular, the diversity of oligosaccharides is strictly controlled by the activity of several enzymes encoded by different glycosyltransferase genes. Thus, the expression of a particular molecular species of glycosphingolipid in cells and tissues can be altered by manipulating the expression of specific glycosyltransferase genes. Cancer cells express glycosphingolipid molecular species that are cancer-specific antigens not found in noncancerous parental cells [6]. The expression of these glycosphingolipids is due to the expression of glycosyltransferase genes encoding specific enzymes that mediate their synthesis in conjunction with the oncogenic transformation of the parent cells.

## 3. Role of Gangliosides in Cellular Function

Although the role of glycosphingolipids in regulating cellular functions is not fully understood due to difficulties in analyzing these molecules, several studies using genetically engineered cells and mice have shown that gangliosides interact with co-localized proteins in cellular membranes and function as modulators (Table 1). In particular, the role of glycosphingolipids as regulators of receptor-type tyrosine kinases has been intensively investigated. For example, monosialoganglioside GM3 regulates signal transduction mediated by epidermal growth factor (EGF) via inhibition of the tyrosine kinase activity of the EGF receptor in the cell membrane [15]. In contrast, disialoganglioside GD3 promotes cellular signal transduction by interacting with several signaling proteins, such as Lyn, transient axonal glycoprotein-1 (TAG-1), p130Cas, and paxillin [6,16,17,18]. Differential expression of various molecular species of gangliosides in each cell type appear to regulate different cellular responses through interactions with membrane proteins specifically expressed in that cell type.

Gangliosides with complex oligosaccharide structures (complex gangliosides) are the primary components in tissues of the central nervous system and modulate the functions of membrane proteins in neurons. For example, ganglioside GM1 modulates signal transduction in neuronal membranes via nerve growth factor receptors to promote neuronal differentiation [21,22]. Furthermore, the polysialylated complex gangliosides GD1b and GT1b exhibit the potential to promote neuronal cell proliferation [23].

Loss of gangliosides, by contrast, perturbs signal transduction through the leptin receptors and insulin receptors expressed in neurons in the hypothalamus [3]. In the forebrain of neuron-specific ganglioside-deficient mice, leptin/leptin receptor signal transduction is attenuated in the hypothalamic neuronal membrane, which results in dysregulation of body energy homeostasis and the development of obesity. Analyses of mice lacking part of the oligosaccharide structure of gangliosides demonstrated that a-series gangliosides (Figure 1) positively regulate the leptin receptor to mediate signaling in hypothalamic neurons [24]. Increased mediobasal hypothalamus insulin signaling suppresses white adipose tissue lipolysis [26]. Insulin-associated signal transduction in hypothalamic neurons is also dysregulated in ganglioside-deficient mice, and the phenotype of these mice indicates that hypothalamic gangliosides modulate insulin receptor signaling and protein levels. The loss of hypothalamic gangliosides enhances insulin signaling and results in a decrease in fasting-induced lipolysis. It was also shown that gangliosides are required for the secretion of leptin from adipocytes into the blood [25]. These findings indicate that decreased expression of gangliosides in hypothalamic neurons and adipocytes promotes the progression of obesity and the onset of diabetes.

In the mammalian immune system, neutral glycosphingolipids containing very-long-chain fatty acids in the ceramide portion function as mediators of inflammatory signaling [27] as well as innate and acquired immunity [11,12,13,28]. As loss of gangliosides has been associated with abnormal immune responses [29], gangliosides containing very-long-chain fatty acids may exhibit similar effects in the mammalian immune system.

## 4. Gangliosides and Disease

Lysosomal disease, a hereditary disease characterized by abnormal accumulation of gangliosides in the tissues, was discovered more than 50 years ago [30], and since then there have been a number of studies examining the relationship between gangliosides and disease (Table 2). In recent years, many membrane proteins that interact with gangliosides have been identified, spurring research into the relationship between these interactions and various diseases. These studies demonstrated that abnormal expression of gangliosides is a molecular basis for the onset of diseases such as diabetes [4], central nervous system diseases [5], and cancer [6].

With regard to diabetes, the effect of GM3 on insulin signaling has been thoroughly investigated [4]. Several experimental models demonstrated that GM3 ganglioside interacts with insulin receptors in the cellular membrane to negatively regulate signal transduction. Abnormal accumulation of GM3 was shown to induce insulin resistance in cells, which is the molecular basis of the pathology of type 2 diabetes [19]. Accumulation of GM3 is triggered by the chronic inflammatory stimulation of cells which is associated with a pathology of type 2 diabetes [20].

Regarding neurologic diseases, humans harboring a mutation in the GM3 synthase gene, which encodes a key enzyme involved in the synthesis of the backbone structure of gangliosides, reportedly develop autosomal recessive infantile-onset symptomatic epilepsy syndrome [31]. Hereditary spastic paraplegia is known to be associated with mutations in the *B4galnt1* gene encoding GM2/GD2 synthase, a rate-limiting enzyme in the synthesis of the complex gangliosides abundant in central nervous system tissues [32]. Genetically engineered mice lacking complex gangliosides also exhibit various neurologic symptoms.

Gangliosides expressed on the cell surface serve as receptors for certain viruses and bacterial toxins and thus mediate infection and cytotoxicity. For example, tetanus toxin enters neurons via gangliosides from the neuromuscular junction [34]. The tetanus toxin molecules are transported to central nervous system tissue via axonal transport, where they disrupt the release of neurotransmitters from neuronal synapses, resulting in spastic paraplegia. Patients with ganglioside storage disease can develop a variety of neurologic disorders [30]. It was suggested that GM1 ganglioside accumulates in senile plaques in Alzheimer’s disease, and these plaques serve as scaffolds for accelerated amyloid-β aggregation, which is the molecular pathology of this disease [33]. These findings indicate that the central nervous system tissues, such as the brain, require appropriate amounts of gangliosides to remain in a healthy condition.

Since certain gangliosides were discovered as cancer-specific antigens [6,13,15,35], additional studies have examined the role of gangliosides specifically expressed in cancer cells. Oncogenic transformation alters the expression levels of glycosyltransferase genes, resulting in upregulated expression of cancer-specific gangliosides. Studies using genetically engineered cells have shown that cancer-specific gangliosides modulate signal transduction involved in cancer growth and metastatic potential.

## 5. Diet and Ganglioside Expression

A number of studies are currently underway examining the effect of diet on the expression level of tissue gangliosides. Although the details remain to be fully elucidated, recent progress has revealed new information regarding the effects of diet on ganglioside dynamics, metabolism, and induction. Cellular responses to nutrients and stress have also been identified as factors involved in the regulation of tissue ganglioside expression. This section describes these findings.

### 5.1. Gangliosides in Foods

Gangliosides are found in animal-derived foods such as meat, fish, egg yolk, and dairy products. The composition of molecular species of gangliosides in these foods are listed in Table 3. Dairy products primarily contain GD3 [36], but meat and fish contain GM3 [37]. These foods also contain complex gangliosides as minor components. Complex gangliosides are major lipid components in animal brains used as food in certain cultures.

GD3 is the major ganglioside in human breast milk during the early lactation stage, whereas GM3 becomes the major ganglioside in later stages [39,40]. Some complex gangliosides are also found in breast milk [40,41,42,43]. Bovine milk contains GD3 as the major ganglioside, and therefore, formula prepared using bovine milk also contains this ganglioside [43]. Gangliosides can remain stable in breast milk for long periods at several storage temperatures [45]. Pasteurization has almost no effect on the stability of gangliosides in breast milk.

Plants and fungi contain almost no sialic acid and therefore do not contain gangliosides. However, edible plants such as soybean, corn, rice, wheat, and konjac (*Amorphophallus konjac*, K. Koch) are rich in glucosylceramide [46,47], a ganglioside precursor. Although not used as a food source, marine sponge (*Agelas mauritianus*) contains a neutral glycosphingolipid, αGalCer, which activates innate immune responses in mammals [28].

### 5.2. Digestion and Absorption of Gangliosides in Foods

**Figure 2:**
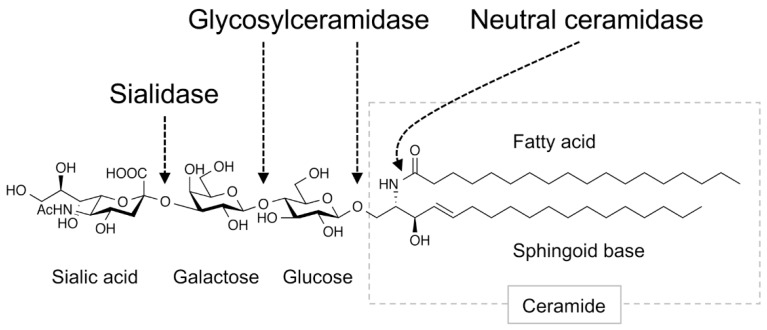
Ganglioside-degradation enzymes in the intestinal tract. Digestive enzymes that degrade gangliosides in the mammalian intestinal tract are shown. Arrows indicate linkage regions in the ganglioside degraded by these enzymes. Figure shows GM3 as an example ganglioside.

Gangliosides contained in foods are degraded in the intestinal tract (Figure 2), and the remaining intact structures are not taken up into the blood and lymph fluid. However, the products of ganglioside degradation, such as saccharides and lipids, are supplied to the tissues, where they promote the biosynthesis of gangliosides. In mammals, gangliosides are first degraded in the small intestine by sialidase/neuraminidase, which removes the sialic acid residues [48], and then sequentially degraded into saccharides and ceramides by glycosylceramidase present in the small intestinal mucosa [49]. Ceramides are further degraded into sphingoid bases and free fatty acids by neutral ceramidase [50,51]. These degradation products (saccharides and lipids) are absorbed by small intestinal epithelial cells and then partially re-synthesized into glycosylceramides [7,49]. Gangliosides are thought to be re-synthesized only in tissue cells that express enzymes involved in ganglioside synthesis. The results of these studies indicate that saccharides and lipids derived from gangliosides induce ganglioside expression in tissues. The sialic acid *N*-glycolylneuraminic acid (Neu5Gc), which is found in edible animals but not in humans, is abundant in red meat and dairy products [52]. Consumption of these foods supplies tissue cells with Neu5Gc and results in the synthesis of ganglioside species containing Neu5Gc, which are known to be highly expressed in cancer cells [53].

Breast milk is a rich source of oligosaccharides containing sialic acid, and the primary oligosaccharide, sialyl lactose, has the same structure as the oligosaccharide of GM3 [8,9]. Infants raised on breast milk have a significantly higher sialic acid content in the gangliosides and glycoproteins in the frontal cortex of the brain than infants fed formula [8]. Sialyl lactose has isomers that exhibit different sialic acid binding patterns, and a model experiment in which pigs were fed these isomers showed that each isomer increases the sialic acid content of gangliosides in a similar manner in several brain regions [9]. These results indicate that the degradation products of sialyl lactose promote ganglioside synthesis in brain tissues. Several animal experiments have shown that a high sialic acid content in gangliosides in the brain is associated with neuronal development, based on the formation and stabilization of functional synapses and neural circuits, leading to improved cognitive function, memory formation, and learning ability [54].

### 5.3. Indirect Effects of Dietary Components on Ganglioside Expression

Several studies have found that ganglioside expression in tissues is affected by various nutrient deficiencies or foods that do not provide components for ganglioside synthesis. A study investigating the effects of undernutrition in neonatal rats induced by feeding the mothers a low-protein diet during lactation showed a marked decrease in brain ganglioside content, along with decreases in body and brain weight [55]. Ganglioside expression in neonatal rat brain is also affected by thiamine and vitamin A deficiency [56]. Chronic consumption of ethanol also reportedly affects the expression of gangliosides in the rat brain [57]. Vitamin K deficiency is known to be associated with cognitive and behavioral perturbations in rats, as well as changes in the expression of gangliosides in the hippocampus, striatal striatum, and prefrontal cortex hippocampus in the rat brain [58]. Another study reported that a high-fat diet decreases glycosphingolipid levels in mouse liver [59], whereas our previous study revealed that ganglioside content in the liver increases in mice fed a high-fat, very-low-carbohydrate ketogenic diet (LCKD) [10,60].

The molecular mechanism underlying indirect induction of ganglioside expression in tissues mediated by dietary components requires further investigation. As considerable research has focused on the molecular mechanism underlying the effects of ketogenic diets on ganglioside expression in tissues, the details will be described in the next section.

### 5.4. Dietary Induction of Tissue Ganglioside Expression via Transcriptional Regulation of Ganglioside Metabolism-Related Genes

Indirect changes in the expression of gangliosides induced by the diet are suggestive of specific changes in the metabolic pathways of ganglioside synthesis and degradation. A number of genes related to ganglioside biosynthesis and metabolism have been identified in recent years, and it has become clear that the expression of gangliosides in tissue cells is regulated by the expression of specific genes, suggesting that transcriptional regulation of ganglioside metabolism-related genes could be targeted by dietary factors that affect tissue ganglioside expression. The molecular species of ganglioside expressed in each type of tissue cell is controlled primarily by the corresponding glycosyltransferase gene. Analyses of the properties of these genes using cultured cells have revealed several targets for modulating ganglioside metabolism, such as transcription factors, signal transduction, and stress responses; controlling these targets thus regulates the expression of gangliosides in cells [1,2].

#### 5.4.1. Induction of Ganglioside Expression in a Cultured Cell Line by Glucose Starvation

We previously found that ganglioside expression was increased in cultured cells in which glucose starvation was induced by treatment with 2-deoxyglucose, an inhibitor of glycolysis [61]. This change in expression was correlated with the expression levels of glycosyltransferase genes involved in ganglioside synthesis, suggesting that glucose starvation induced ganglioside expression in these cells via transcriptional control of the related genes. A similar stress response was found to control transcription of the gene encoding the glycosyltransferase that catalyzes synthesis of intracellular glycoproteins [62,63]. Thus, we speculated that this is a conserved response to the effects of glucose starvation on the synthesis of glycosphingolipids and glycoproteins in mammalian cells. However, whether a similar response occurs in tissue cells in vivo in response to dietary interventions remains unclear. Our study using cultured cells also showed that levels of neutral glycosphingolipids decreased due to downregulation of the expression of related genes [61,64], indicating that the balance between intracellular metabolism of gangliosides and neutral glycosphingolipids also affects the increased expression of gangliosides brought about by glucose starvation.

#### 5.4.2. Induction of Tissue Ganglioside Expression in Model Mice Fed a Very-Low-Carbohydrate Ketogenic Diet

Responses similar to those in cells treated with 2-deoxyglucose can be induced in mice fed a LCKD [65] developed for its antiepileptic effects. We therefore established an LCKD mouse model [66] and analyzed the effect of the LCKD on gene expression in tissues using gene expression profiling [10]. LCKDs are characterized by a specific nutritional balance of reduced glucose content and increased fat content. LCKDs have been shown to suppress even intractable epilepsy resistant to currently available antiepileptic drugs, and they have been covered by insurance in Japan since 2016. In addition, recent studies reported beneficial effects of LCKDs in patients with various diseases, including obesity, type 2 diabetes mellitus, autism, cardiomyopathy, neurodegenerative diseases, and cancer [67]. The lipid components of an LCKD are degraded in the liver, and ketone bodies are produced. These ketone bodies are supplied to the tissues as a nutrient in substitution of glucose and used in brain tissues as the only energy source that can be used instead of glucose [68]. In lactating infants, ketone bodies serve as the major nutrient, instead of glucose. We designed an LCKD model using obese mutant mice (*ob*/*ob*) to evaluate the diet’s effect based on the hyperphagic phenotype of the mice [66]. Furthermore, using *ob*/*ob* mice is advantageous because it is easy to detect an increase of hepatic ganglioside expression because the levels of gangliosides in the liver are lower than in wild-type mice [60,69].

**Figure 3:**
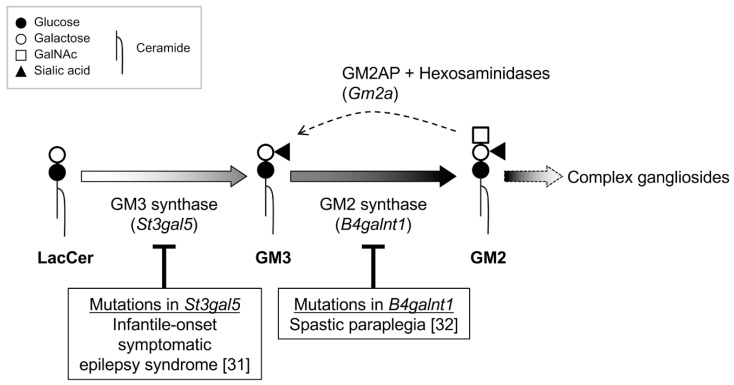
Neurologic disorders associated with mutations in ganglioside synthases. Schematic illustration of the relationship between ganglioside synthesis and mutations in the *St3gal5* and *B4galnt1* genes found in infantile-onset symptomatic epilepsy syndrome [31] and spastic paraplegia [32]. Ganglioside GM2 is degraded to GM3 by lysosomal hexosaminidases in concert with GM2 activator protein (GM2AP) (encoded by *Gm2a*) in lysosomes.

A gene expression microarray analysis was conducted using hepatic total RNA from mice fed the LCKD for seven weeks. Numerous genes exhibiting altered expression compared with mice fed regular chow were detected [10,67,70]. As expected, genes involved in the synthesis of glycan for gangliosides and glycoproteins exhibited significantly altered transcription in the LCKD-fed mice [10]. Several glycosyltransferase genes involved in ganglioside synthesis were found to be significantly upregulated, including GM3 synthase (*St3gal5*) and GM2 synthase (*B4galnt1*), a lack of which has been linked to hereditary epilepsy [31] and spastic paraplegia, respectively [32] (Figure 3). Furthermore, the expression of *Gm2a*, which encodes the lysosomal GM2 activator protein (GM2AP) required for ganglioside degradation, was also downregulated. These changes suggested that the LCKD increased the levels of gangliosides in the liver. Indeed, the increased ganglioside content in the liver of LCKD-fed mice correlated with the changes in gene expression [10]. The ganglioside content in the liver was more than three times higher than that of chow-fed mice. Furthermore, a similar increase was also observed in serum gangliosides, which are derived from liver gangliosides. An analysis of the ganglioside content in the LCKD used in this study (Bio-Serve F3666) showed that this diet contains GD3 as a major component and GM3 as a minor component. However, GD3 was not detected among the gangliosides in the liver and serum of the mice used in the experiment [10,60], indicating that the gangliosides in the LCKD did not directly affect the expression of gangliosides in the liver and serum of the LCKD-fed mice. The gangliosides contained in the F3666 are mainly derived from butter, a primary ingredient of this diet.

These results and our previous findings indicate that the LCKD indirectly increased tissue expression of gangliosides in mice. As the glycosphingolipid content in the liver is known to decrease in *ob*/*ob* mice fed a high-fat diet [59], we conclude that the low-carbohydrate or ketogenic properties of the LCKD induce tissue ganglioside expression. Elucidating the precise molecular mechanism underlying the ganglioside-inducing effects of LCKDs is a subject of our future research. As the expression levels of ganglioside metabolism-related genes are altered in LCKD-fed mice, we will focus on LCKD-mediated transcriptional regulation of these genes.

In brain tissue, a dramatic induction of expression and changes in the structure of gangliosides are observed from the embryonic period to lactation. Ketone bodies derived from the placenta and breast milk are the major nutrients for the fetus and infants [68]; thus, these may significantly affect the changes in ganglioside expression and structure observed during this period. In contrast, fatty liver and hyperglycemia, the major phenotypes of *ob*/*ob* mice, are improved by LCKD feeding [66,67]. As increased expression of gangliosides in the liver and serum are correlated with improvements in these pathologies, and gangliosides may play a contributory role.

Long-term LCKD feeding was also shown to affect the glycosylation status of liver glycoproteins [70]. These structural changes in liver glycoproteins were not correlated with the expression levels of glycosyltransferase genes but were correlated with downregulated expression of genes involved in the synthesis of sugar nucleotide donors for protein glycosylation in the liver. This property differed from glycosphingolipid metabolism in the liver of LCKD-fed mice. In other words, the expression levels of liver gangliosides were not correlated with the expression levels of genes involved in the synthesis of the sugar nucleotide donors but were correlated with that of glycosyltransferase genes. These results indicate that ganglioside expression depends more on the expression levels of glycosyltransferase genes than on that of genes involved in the synthesis of sugar nucleotide donors. Glycosphingolipids are minor glycoconjugate components compared to glycoproteins in the liver, and the number of donors is presumed to have minimal impact on the synthesis of glycosphingolipids. Differences in the kinetic properties of glycosyltransferases for donors may also affect the production of glycosphingolipids and glycoproteins.

#### 5.4.3. Effect of LCKD Feeding on Brain Gangliosides

Gangliosides and their metabolism-related genes regulated by LCKD feeding include those associated with hereditary epilepsy. Gangliosides are expressed primarily in central nervous system tissues, where LCKD feeding exhibits antiepileptic effects. These observations led us to speculate that LCKDs affect the expression of gangliosides in central nervous system tissues. Gene expression analyses in the hippocampus and cerebral cortex, which are considered primary epileptogenic foci, indicated that the effect of LCKDs on gene expression in these tissues is weaker than that in the liver [10]. Among ganglioside metabolism-related genes, we found a significant decrease in the expression level of only *Gm2a*, as observed in the liver. However, the level of decrease was smaller than that in the liver, and the ganglioside content in these tissues was unchanged by LCKD feeding.

*Gm2a*-deficient mice exhibit an accumulation of gangliosides in certain brain regions, such as the piriform, entorhinal cortex, amygdala, hypothalamic nuclei, and cerebellum [71]. Thus, we analyzed the effect of LCKD feeding on ganglioside expression in the cerebellum and found that levels of complex gangliosides were significantly increased in LCKD-fed mice [10]. This result indicates that LCKDs affect ganglioside expression in a limited area of central nervous system tissues susceptible to *Gm2a*.

LCKDs have been shown to suppress refractory epilepsy, which cannot be controlled with currently available drugs [72]. However, the molecular basis of the antiepileptic effect of LCKDs has yet to be fully elucidated. Recent studies have shown that LCKDs exert pleomorphic effects relevant to the nervous system. For example, LCKDs suppress epilepsy by increasing levels of the inhibitory neurotransmitter γ-aminobutyric acid (GABA) [73] or by inhibiting lactate dehydrogenase (LDH) activity in astrocytes and neurons [74]. However, LCKDs appear to have further as yet unidentified effects, as they suppress epilepsy that cannot be controlled with drugs that regulate GABA and LDH.

The GM3 synthase encoded by *St3gal5* initiates ganglioside synthesis, and a deficiency in this enzyme results in deficiencies in most gangliosides in vivo [31,75]. In humans, loss of function of the GM3 synthase due to mutations in *St3gal5* results in infantile-onset symptomatic epilepsy syndrome [31]. As *St3gal5* encodes a sialyltransferase specific for GM3 synthesis and no other function for this protein has been identified, the symptoms of this condition can be attributed to ganglioside deficiency. Loss of function of the GM2 synthase due to mutations in *B4galnt1* results in hereditary spastic paraplegia [32]. The complex gangliosides are thought to contribute to the formation of synaptic membranes and regulate neurotransmission via neurotransmitters due to their negative charges [54,76,77]. We hypothesize that this property is the molecular basis of the antiepileptic effect of LCKD-induced increased complex ganglioside expression in central nervous system tissue. These observations indicate that increasing the levels of complex gangliosides could be an effective means of protecting against the onset of neurologic diseases. Decreased expression of *Gm2a* can also contribute to this effect; however, excessive accumulation of gangliosides due to *Gm2a* deficiency is also associated with the symptoms and onset of epilepsy [78], indicating that modest transcriptional repression of *Gm2a*, as we observed in LCKD-fed mice, is important. Although any relationship between the findings of this study and epilepsy are purely speculative at present, we believe that these data will contribute to future studies of treatments for epilepsy.

## 6. Conclusions

Progress in biochemistry, molecular biology, nutrition, food science, and glycobiology research has clarified the effects of diet on ganglioside expression in vivo. The scientific studies discussed in this review demonstrated that dietary intake of foods containing gangliosides and its raw materials and dietary interventions to alter the expression of ganglioside metabolism-related genes are means of controlling ganglioside expression in vivo. For continued progress in this field, more information will be required regarding what foods contain gangliosides and the components necessary for their synthesis. Although a number of studies have clarified the mechanism of transcriptional regulation of ganglioside metabolism-related genes in cells, a new approach will be required from the viewpoint of dietary interventions. As little research has been conducted on tissue expression of gangliosides using diet-controlled model animals such as LCKD-fed mice, this will be a major focus of continued research. Many questions remain unanswered in this field; thus, additional studies will be needed to treat and prevent ganglioside-related diseases.

## Figures and Tables

**Table 1:** Gangliosides and their interacting proteins.

Ganglioside	Interacting Protein	Reference
GM3	EGF receptor	[15]
	Insulin receptor	[4,19,20]
GD3	p130Cas, paxillin	[18]
	Lyn, TAG-1	[16,17]
GM1, GD1a, GD1b, GT1b	NGF receptors	[21,22,23]
GM1, GD1a	Leptin/Ob receptor	[3,24]
b-series gangliosides	Leptin	[25]

EGF, epidermal growth factor; TAG-1, transient axonal glycoprotein-1; NGF, nerve growth factor.

**Table 2:** Ganglioside-related diseases.

Ganglioside	Related Disease	Reference
GM3	Type 2 diabetes mellitus	[4]
	Epilepsy	[31]
GM2	GM2 gangliosidosis	[30]
	Spastic paraplegia	[32]
GM1	GM1 gangliosidosis	[30]
	Alzheimer’s disease	[33]
GM1, GD1a	Obesity	[3,26]
GD3, GD2	Melanoma, other cancers	[6]

**Table 3:** Ganglioside components in foods.

Food		Major (Minor) Ganglioside Component	Reference
Meat	Beef	GM3 (GD3, GD1a)	[37]
	Pork	GM3 (GD3, GD1a, GD1b)	[37]
	Chicken breast	GM3 (GD3)	[37]
	Chicken thigh	GM3 (GD3, GD1a, GD1b)	[37]
Fish	King salmon	GM3 (GD3)	[37]
	Snapper	GM3 (GD3, GD1a, GD1b)	[37]
	Island mackerel	GM3 (GD3, GD1a, GD1b)	[37]
	Turbot	GM3 (GD3, GD1a, GD1b, GT1b)	[37]
	Tuna	n.d.	[38]
Egg yolk		n.d.	[38]
Human breast milk	Early stage	GD3	[39,40]
	Late stage	GM3	[39,40]
	All stages	(GM1 and others)	[40,41,42]
Dairy products	Bovine milk	GD3 (GM3)	[43]
	Bovine butter	GD3 (GM3)	[36]
	Yogurt	n.d.	[38]
	Infant formula	GD3 (GM3)	[43,44]
	Cheddar cheese	n.d.	[38]
	Whey	GD3 (GM3)	[44]
LCKD (Bio-Serv F3666)		GD3 (GM3)	This article

n.d., not determined; LCKD, low-carbohydrate ketogenic diet.

## Abbreviations

GSLs	glycosphingolipids
Neu5Ac	*N*-acetylneuraminic acid
Neu5Gc	*N*-glycolylneuraminic acid
EGF	Epidermal growth factor
TAG-1	transient axonal glycoprotein-1
NGF	nerve growth factor
LCKD	low-carbohydrate ketogenic diet
*ob*/*ob*	obese mutant
GM2AP	GM2 activator protein
GABA	γ-aminobutyric acid
LDH	lactate dehydrogenase
